# COVID-19 in people aged 18–64 in Sweden in the first year of the pandemic: Key factors for severe disease and death

**DOI:** 10.1016/j.gloepi.2022.100095

**Published:** 2022-11-24

**Authors:** Annika Rosengren, Mia Söderberg, Christina E. Lundberg, Martin Lindgren, Ailiana Santosa, Jon Edqvist, Maria Åberg, Magnus Gisslén, Josefina Robertson, Ottmar Cronie, Naveed Sattar, Jesper Lagergren, Maria Brandén, Jonas Björk, Martin Adiels

**Affiliations:** a Department of Molecular and Clinical Medicine, Institute of Medicine, Sahlgrenska Academy, University of Gothenburg, Gothenburg, Sweden; b Region Västra Götaland, Department of Medicine Geriatrics and Emergency Medicine, Sahlgrenska University Hospital Östra Hospital, Gothenburg, Sweden; c Occupational and Environmental Medicine, School of Public Health and Community Medicine, Institute of Medicine, Sahlgrenska Academy, University of Gothenburg, Gothenburg, Sweden; d School of Public Health and Community Medicine, Institute of Medicine, Sahlgrenska Academy, University of Gothenburg, Gothenburg, Sweden; e Region Västra Götaland, Regionhälsan, Gothenburg, Sweden; f Region Västra Götaland, Department of Infectious Diseases, Sahlgrenska University Hospital, Gothenburg, Sweden; g Department of Infectious Diseases, Institute of Biomedicine, Sahlgrenska Academy, University of Gothenburg, Gothenburg, Sweden; h Department of Mathematical Sciences, Chalmers University of Technology and University of Gothenburg, Gothenburg, Sweden; i Institute of Cardiovascular and Medical Sciences, University of Glasgow, Glasgow, United Kingdom; j Upper Gastrointestinal Surgery, Department of Molecular Medicine and Surgery, Karolinska Institutet, Karolinska University Hospital, Sweden; k School of Cancer and Pharmaceutical Sciences, King's College London, United Kingdom; l Stockholm University Demography Unit (SUDA), Department of Sociology, Stockholm University, Stockholm, Sweden; m Institute for Analytical Sociology (IAS), Linköping University, Norrköping, Sweden; n Division of Occupational and Environmental Medicine, Lund University, Lund, Sweden; o Clinical Studies Sweden, Forum South, Skåne University Hospital, Lund, Sweden

**Keywords:** COVID-19, Mortality, Intensive care, Population study, Occupation, Comorbidity

## Abstract

**Background:**

Studies on risk factors for severe COVID-19 in people of working age have generally not included non-working persons or established population attributable fractions (PAFs) for occupational and other factors.

**Objectives:**

We describe the effect of job-related, sociodemographic, and other exposures on the incidence, relative risks and PAFs of severe COVID-19 in individuals aged 18–64.

**Methods:**

We conducted a registry-based study in Swedish citizens aged 18–64 from 1 January 2020 to 1 February 2021 with respect to COVID-19-related hospitalizations and death.

**Results:**

Of 6,205,459 persons, 272,043 (7.5%) were registered as infected, 3399 (0.05%) needed intensive care, and 620 (0.01%) died, with an estimated case fatality rate of 0.06% over the last 4-month period when testing was adequate. Non-Nordic origin was associated with a RR for need of intensive care of 3·13, 95%CI 2·91–3·36, and a PAF of 32·2% after adjustment for age, sex, work, region and comorbidities. In a second model with occupation as main exposure, and adjusted for age, sex, region, comorbidities and origin, essential workers had an RR of 1·51, 95%CI, 1·35–1·6, blue-collar workers 1·18, 95%CI 1·06–1·31, school staff 1·21, 95%CI 1·01–1·46, and health and social care workers 1·89, 95%CI 1·67–2·135) compared with people able to work from home, with altogether about 13% of the PAF associated with these occupations. Essential workers and blue-collar workers, but no other job categories had higher risk of death, adjusted RRs of 1·79, 95%CI 1·34–2·38 and 1·37, 95%CI 1·04–1·81, with adjusted PAFs of altogether 9%.

**Conclusion:**

Among people of working age in Sweden, overall mortality and case fatality were low. Occupations that require physical presence at work were associated with elevated risk of needing intensive care for COVID-19, with 14% cases attributable to this factor, and 9% of deaths.

## Introduction

While SARS-CoV-19-2 infection can occur at any age, severe COVID-19 complications, and death are uncommon or rare among the young or middle-aged [1]. In an early study [2], only a small proportion of all COVID-19 deaths in selected locations in Europe, Canada, and the United States occurred among people <65 years, although this age group represents most of the population. Still, although absolute risks of severe COVID-19 and death in the population are low in this age group, the spread of SARS-CoV-2 during the pandemic has resulted in large numbers of deaths and hospitalisations among people <65 years [3].

In older persons, comorbidities and need of assisted living (home care or living in a long-term care facility) play a major role for severe outcomes, including death, with over 55% of all COVID-19 deaths among people aged 55 and older in Sweden attributable to assisted living [4]. Yet, although comparatively healthy younger and middle-aged adults form a large proportion of hospitalised cases and of those needing ventilatory support [3] less is known about this group, although a focus on occupational exposures will automatically target younger subgroups. In a recent Swedish cohort study, foreign-born workers in essential occupations had the highest risk for being infected, hospitalised, or needing intensive care, while Swedish-born workers in non-essential occupations had the lowest risk [5]. Still, many severe COVID-19 cases and deaths in younger age groups will have occurred among those not working, because of early retirement, unemployment, and other reasons, with working people likely to be healthier than those not working. Recently, a Danish study found that socially marginalised and psychiatrically vulnerable individuals who were infected at a mean age of 40 years had substantially elevated risks of adverse health outcomes following SARS-CoV-2 infection compared to infected without these characteristics [6].

The overall morbidity and mortality risk of COVID-19 attributable to comorbidities, occupations, and sociodemographic factors have not been extensively studied among adults <65 years. In particular, the relative contribution of occupational, health-related, and sociodemographic factors to severe COVID-19 manifestations expressed as population attributable fractions (PAFs) has not been systematically assessed. Data on PAFs added to estimates of absolute and relative risk are important to allocate resources and efforts where they will be most useful. In the present report, we sought to estimate factors of importance for being seriously ill or die associated with COVID-19 in all Swedish citizens aged 18–64 years during the first year of the pandemic.

## Methods

### Data sources

In this population-based cohort study we used data from nationwide Swedish registries. The Total Population Register held by Statistics Sweden (SCB) registers all citizens from data kept by the Swedish Tax Agency and was used to identify all citizens in Sweden alive on 1 January 2020.

Health care in Sweden is organized by the 21 Swedish counties or regions and is almost exclusively publicly financed, at low cost to the individual. The Swedish National Board of Health and Welfare (NBHW) records all hospitalisations, hospital outpatient visits, and deaths in Sweden in the nationwide Patient and Cause of Death Registers [7,8]. Diagnoses are coded according to the International Classification of Diseases, 9th revision (ICD-9) (1987–1996) and 10th revision (ICD-10) from 1997 and onwards. See Supplement p. 2 and tables S1–S2 for the definition of COVID-19 and other codes used for this study.

Data on admissions into intensive care units (ICU) were collected through the Swedish Intensive Care Registry (https://www.icuregswe.org/en/) with complete coverage of all patients with COVID-19 intensive care in Sweden. High-flow oxygen therapy was also administered through high dependency units not covered by Intensive Care Registry and identified through hospital codes; these cases were also defined as ICU care.

SmiNet, a registry for communicable disease surveillance in Sweden kept by the Swedish Public Health Agency, stores electronic reports on positive positive polymerase chain reaction (PCR) tests on COVID-19 infections. PCR testing was restricted during the first months of the pandemic. Negative tests are not reported.

Care of the elderly is governed by law and carried out by the municipalities (lower-level local government entities of which there are 290 in Sweden). The municipality can provide a subsidized home-help service, sometimes several times per day and at night, if needed, or provide a place in a long-term care facility. Although care is publicly financed, many of these facilities are privately owned. The NBHW keeps a registry of municipal care that registers public and private home care and care in long-term care facilities [9].

Statistics Sweden keeps registries on education and housing, for example on living space area, number of persons living in the household, and population density of residence, with the latter based on different spatial aggregation scales, with the smallest scale corresponding to Demographical Statistical Areas (DeSo) zones which are a type of demographic area. Sweden is divided into 5984 DeSo zones of varying size, where each zone, typically inhabited by 700–2700 people, is encoded according to its degree of urbanisation (urban, suburban, or rural).

### Sweden's approach to the pandemic

The approach to COVID-19 in Sweden differed from that of other countries in that there were mostly voluntary restrictions and no formal lock-down. Early on during the pandemic the Public Health Agency in Sweden issued a ban on visits to nursing home and on large congregations, together with recommendations directed to older and to vulnerable people to avoid face-to-face contacts. Schools up to lower secondary school (grade7–9) stayed open. Students from upper secondary school and higher were mainly taught online. There were general recommendations to work from home, if possible.

### Study population

All persons registered as living in Sweden aged 18–64 years, alive on 1 January 2020 (59% of the Swedish population aged ≥18) were included in the study, except for a small number (<0.5%) with reused personal identity numbers (PINs).

### Definitions

Statistics Sweden provided sociodemographic data updated until 2019. Country of origin was dichotomised into those born in a Nordic country (Sweden, Denmark, Finland, Iceland, or Norway) and those born in any other country, representing diverse ethnic groups and nationalities, with Western Asia/Middle East and Eastern Europe being the most common, followed by East Africa. Data on single countries of origin are not provided by Statistics Sweden. Additional sociodemographic variables included housing (m^2^ per habitant, number of people per household); residing in an urban, suburban, or rural area; population density of residential area; annual household income divided by the number of members in the household; formal education defined as compulsory only (≤9 years), mid-range education (10–12 years), or college/university education.

We defined 11 mutually exclusive individual exposure variables: 1) university students 2) a reference category comprising all persons in occupations where it was possible to work from home or with few social contacts, 3) not working (mostly unemployed or homemaker), 4–7) four occupational categories (defined below and in table S3), 8) retired, 9) early retirement for a medical reason, 10) receiving home care, and 11) living in a long-term care facility. The latter two categories were defined by data in the registry of municipal care [9].

Occupation was registered as a four-digit occupational code according to the Swedish Standard Occupational Classification (table S3), which is the Swedish adaptation of the International Standard Classification of Occupation (ISCO-08). Two separate groups were defined for a) school and preschool staff and b) persons employed in health and social care. Other essential workers were grouped together; among these, service sector workers, police and security services, postal and delivery workers, transport workers. Blue-collar occupations not included among the previously described categories, but which required people to work on-site formed a separate category.

For individuals without a valid occupational code, we used an income variable (main source of income) to separate those drawing early statutory pension (available from the age of 62 years) from early retirement due to a medical reason. Early retirement was defined as receiving disability benefits because of physician-certified medical reasons. University students most often restricted to online studies during the pandemic were defined separately by university registration or by having student loans as their main income source.

Baseline comorbidities (table S1) were collected from the National Patient Register with very good coverage for major events such as acute myocardial infarction and stroke, but less so for diagnoses managed in primary care, if uncomplicated, such as hypertension or diabetes [8].

### Outcomes

We defined four outcomes as 1) infection with COVID-19 based on a positive PCR test, including all cases specified below, 2) severe COVID-19 defined as any hospitalisation or death with a diagnosis of COVID-19 (ICD-10 code U071 and U072), 3) intensive care related to COVID-19, and 4) death with a COVID-19 diagnosis as an underlying or contributory cause of death. To determine death from COVID-19, the underlying cause had to be related to symptoms or complications associated with COVID-19 (table S2). We used a prespecified algorithm to exclude hospitalisations and deaths due to other causes, in which cases a diagnosis of COVID-19 was incidental.

### Statistical methods

Outcomes were recorded up to 1 February 2021, with comorbidities and demographic factors recorded until 1 January 2020, to avoid bias due to added reporting during the pandemic.

Case fatality rates were calculated as the ratio of deaths divided by cases. Because few PCR tests were carried out during the first wave in the spring, an unknown number of symptomatic cases could not be identified. During the second wave, from 1 October 2020 to 1 February 2021, when testing of all suspected cases was recommended, case fatality rates were calculated as number of deaths divided by cases.

Baseline data are reported without imputation. Except for education (4·9%) missing data were < 1%. Missing information on living conditions (home care, long term care facility) was labelled as independent living before imputation, whereas other missing data were imputed using Multivariate Imputation by Chained Equations (MICE) [10] with five iterations. Variables included in the imputation algorithm were: age, sex, Nordic origin, area per habitant, number of habitants in household, household type, education, underlying medical conditions, income per habitant, population density in region, type of region (urban, rural, suburban) and occupation.

Results are presented from one imputed dataset. Prior analyses showed little benefit of using more than one imputed dataset [4].

Poisson regression models, with the total population of this study as denominator, were used to estimate risk ratios (RRs). 95% confidence intervals (CI) were calculated using the adjusted method by Zou [11].

To assess the major risk exposures for COVID-19, and their contribution to population risk, three models were developed: 1) separate univariate models tested risk ratios for each variable alone (table S4); 2) model with occupation as main exposure, adjusted for confounders (age, sex, region, comorbidities and Nordic origin) (Fig. S1) 3) Model with Nordic origin as main exposure, adjusted for confounders (age, sex) and mediators (region, comorbidities, occupation) (Fig. S2).

Population attributable fraction (PAF), defined as the proportion of all cases of a particular outcome in a population that is attributable to a specific exposure, was calculated using the R package ‘AF’ [12], which allows for confounder adjusted estimation of PAF. Briefly, point estimates are calculated from the predicted number of outcomes under the counterfactual (ie setting all “Diabetes” cases to “No diabetes”) and factual data sets. Confidence intervals were calculated by the sandwich method [13].

### Ethical considerations

Personal identifiers were removed after linkage of the registries and replaced by a code. Because pseudonymised data were used, written informed consent was not applicable. The project was approved by the Swedish Ethical Review Authority (2020–02019).

## Results

### COVID-19 outcomes in the population

Of 18,907 hospitalisations with a diagnosis of COVID-19, 15,783 (83%) had COVID-19 as the main diagnosis, 452 (2%) had a COVID-19-related main diagnosis and COVID-19 as a contributory diagnosis and 2672 (14%) had a main diagnosis that was not related to COVID-19, the latter were not defined as COVID-19 hospitalisations. During the study period, accordingly, among a total of 6,205,459 persons aged 18–64 years, there were altogether 458,317 (7.39%) cases, 16,235 (0.26%) hospitalised, 3399 (0.05%) who needed intensive care and 16,362 (0.26%) who had severe COVID-19 (hospitalisation or death) (Table 1). There were 620 (0.01%) COVID-19 deaths, of which most (80.5%) occurred in a hospital. During the second wave, from 1 October 2020 to 1 February 2021, when testing capacity was adequate, the overall case fatality rate was 0.06%, ranging from 0.01% among persons aged 18–44 years to 0.27% among those aged 55–64 years. Of all hospitalised cases, 3.0% died, and among persons in intensive care, 9.6% died.

**Table 1:** COVID-19 in all Swedish citizens aged 18–64 years: total testing positive for SARS-Cov2, hospitalised, intensive care, and deaths, by age, until 2021-02-01.

	Total population	Age group		
		18 to 44	45 to 54	55 to 64
Population	6,205,459	3,626,975	1,352,792	1,225,692

Total COVID-19 cases				
Cases	458,317 (7.39)	272,043 (7.50)	107,419 (7.94)	78,855 (6.43)
Hospitalised^a^	16,235 (0.26)	4323 (0.12)	4920 (0.36)	6992 (0.57)
ICU^b^	3399 (0.05)	633 (0.02)	1005 (0.07)	1761 (0.14)
Severe COVID-19^c^^d^	16,362 (0.26)	4341 (0.12)	4952 (0.37)	7069 (0.58)
Deaths (%)	620 (0.010)	63 (0.002)	134 (0.010)	423 (0.035)

Case fatality (deaths/cases)				
Case fatality, overall, %	0.14	0.02	0.12	0.54
Case fatality, ^2nd^ period, %	0.06	0.01	0.05	0.27
Hospital case fatality; %	493 (3.04)	45 (1.04)	102 (2.07)	346 (4.95)
ICU case fatality, %	325 (9.56)	29 (4.58)	71 (7.06)	225 (12.78)

Location of death with COVID-19				
At home	76 (12.26)	10 (15.87)	24 (17.91)	42 (9.93)
At a long-term care facility	19 (3.06)	0 (0)	2 (1.49)	17 (4.02)
In hospital	499 (80.48)	49 (77.78)	98 (73.13)	352 (83.22)
Other	26 (4.19)	4 (0)	10 (0.01)	12 (0.02)

Data are n (%). Cases are defined as testing positive, hospitalisation, and/or death.

Abbreviations: ICU: Intensive care unit.

a With an acceptable main diagnosis. List of acceptable main diagnosis is presented in **Table S2.**

b ICU is reported as main diagnosis only; includes high-flow oxygen at high dependency unit.

c Severe COVID-19: hospitalisation or death.

d Case fatality is calculated during period 2 (2020−10−01−2021−02−01).

### Baseline characteristics

Among the total adult population < 65 years, the majority (58.4%) were younger than 45 years, yet only 18.6% and 10.2% among those requiring intensive care or who died, respectively, were in this age group (Table 2). Male sex, non-Nordic origin, low education, and living in an urban area were all more common among individuals with severe outcomes and deaths. Of those who died, one in six, or 16.9% either received home care or lived in a long-term care facility.

**Table 2:** Population characteristics by COVID-19 categories.

	Total population	Non COVID-19 population	Any COVID-19^a^	Severe COVID-19^b^	Intensive care	Death from COVID-19
Number of individuals	6,205,459	5,747,142	458,317	16,362	3399	620

Sociodemographic						
Age (years), mean (SD)	40.8 (13.2)	40.8 (13.2)	40.2 (13.0)	50.2 (10.8)	52.5 (9.7)	55.6 (8.7)

Age group						
18 to 44	3,626,975 (58.4)	3,354,932 (58.4)	272,043 (59.4)	4341 (26.5)	633 (18.6)	63 (10.2)
45 to 54	1,352,792 (21.8)	1,245,373 (21.7)	107,419 (23.4)	4952 (30.3)	1005 (29.6)	134 (21.6)
55 to 64	1,225,692 (19.8)	1,146,837 (20.0)	78,855 (17.2)	7069 (43.2)	1761 (51.8)	423 (68.2)
Male sex, n (%)	3,180,116 (51.2)	2,965,704 (51.6)	214,412 (46.8)	10,044 (61.4)	2405 (70.8)	448 (72.3)
Born in a Nordic country^c^	4,719,503 (76.1)	4,379,843 (76.2)	339,660 (74.1)	8662 (52.9)	1784 (52.5)	378 (61.0)

Residence						
Population density (n/100,000)	3.8 (6.5)	3.8 (6.5)	4.1 (6.6)	4.6 (6.7)	4.7 (6.7)	4.6 (6.3)
Urban	4,703,183 (77.5)	4,330,540 (77.1)	372,643 (81.7)	13,824 (85.1)	2882 (85.3)	538 (87.2)
Rural	881,353 (14.5)	832,450 (14.8)	48,903 (10.7)	1454 (8.9)	294 (8.7)	48 (7.8)
Suburban	486,638 (8.0)	452,080 (8.1)	34,558 (7.6)	975 (6.0)	204 (6.0)	31 (5.0)

Living conditions						
Number of habitants	3.0 (1.7)	3.0 (1.7)	3.2 (1.7)	3.2 (2.1)	3.2 (2.0)	2.8 (3.0)
Area per family member (m^2^)	39.3 (23.0)	39.5 (23.1)	37.0 (20.8)	37.9 (22.7)	38.3 (22.2)	43.3 (25.5)
Annual income/ family member (1000 KSEK)	2.2 (3.9)	2.2 (3.8)	2.3 (5.2)	2.1 (2.6)	2.2 (2.5)	1.9 (1.3)

Education in 2020^d^						
≤ 9 years	868,626 (14.0)	808,572 (14.1)	60,054 (13.1)	3381 (20.7)	754 (22.2)	155 (25.0)
10–12 years	2,614,101 (42.1)	2,412,485 (42.0)	201,616 (44.0)	7162 (43.8)	1521 (44.7)	278 (44.8)
College/ university	2,416,324 (38.9)	2,229,634 (38.8)	186,690 (40.7)	5237 (32.0)	1003 (29.5)	138 (22.3)

Underlying medical conditions						
Obesity (diagnosis)	141,102 (2.3)	128,819 (2.2)	12,283 (2.7)	843 (5.2)	191 (5.6)	54 (8.7)
Hypertension	165,741 (2.7)	154,095 (2.7)	11,646 (2.5)	2016 (12.3)	507 (14.9)	167 (26.9)
Diabetes	140,153 (2.3)	129,407 (2.3)	10,746 (2.3)	1837 (11.2)	478 (14.1)	147 (23.7)
COPD⁎	36,212 (0.6)	33,830 (0.6)	2382 (0.5)	435 (2.7)	92 (2.7)	48 (7.7)
Malignancy	66,771 (1.1)	61,895 (1.1)	4876 (1.1)	406 (2.5)	80 (2.4)	39 (6.3)
Myocardial infarction	33,223 (0.5)	31,014 (0.5)	2209 (0.5)	369 (2.3)	102 (3.0)	33 (5.3)
Stroke	31,392 (0.5)	29,445 (0.5)	1947 (0.4)	285 (1.7)	63 (1.9)	29 (4.7)
Heart failure	18,854 (0.3)	17,651 (0.3)	1203 (0.3)	328 (2.0)	79 (2.3)	41 (6.6)
Atrial fibrillation	36,543 (0.6)	33,888 (0.6)	2655 (0.6)	382 (2.3)	75 (2.2)	32 (5.2)
VTE⁎	69,517 (1.1)	64,101 (1.1)	5416 (1.2)	601 (3.7)	136 (4.0)	40 (6.5)
Dementia	3710 (0.1)	3443 (0.1)	267 (0.1)	66 (0.4)	8 (0.2)	22 (3.5)

Exposure level^e^						
Student	460,360 (7.4)	434,181 (7.6)	26,179 (5.7)	420 (2.6)	66 (1.9)	4 (0.6)
Reference category	2,330,318 (37.6)	2,171,952 (37.8)	158,366 (34.6)	4527 (27.7)	923 (27.2)	126 (20.3)
Essential workers	794,441 (12.8)	731,602 (12.7)	62,839 (13.7)	2227 (13.6)	499 (14.7)	76 (12.3)
Blue-collar workers	898,127 (14.5)	838,838 (14.6)	59,289 (12.9)	2218 (13.6)	552 (16.2)	87 (14.0)
School/preschool staff	374,403 (6.0)	337,626 (5.9)	36,777 (8.0)	856 (5.2)	134 (3.9)	18 (2.9)
Health and social care	605,609 (9.8)	526,638 (9.2)	78,971 (17.2)	2308 (14.1)	405 (11.9)	34 (5.5)
Not working	525,594 (8.5)	499,087 (8.7)	26,507 (5.8)	2084 (12.7)	432 (12.7)	70 (11.3)
Early retirement	164,920 (2.7)	158,475 (2.8)	6445 (1.4)	1124 (6.9)	269 (7.9)	94 (15.2)
Retired	16,039 (0.3)	15,380 (0.3)	659 (0.1)	61 (0.4)	13 (0.4)	6 (1.0)
Home care	16,050 (0.3)	15,185 (0.3)	865 (0.2)	308 (1.9)	59 (1.7)	53 (8.5)
Long-term care facility	19,598 (0.3)	18,178 (0.3)	1420 (0.3)	229 (1.4)	47 (1.4)	52 (8.4)

Data are n (%) or mean (SD).

⁎ Abbreviations: COPD: Chronic obstructive pulmonary disease VTE: venous thromboembolism.

a Any COVID-19 includes all infections, hospitalisations and deaths.

b Severe COVID-19 includes hospitalisation and deaths.

c Missing data for born in Nordic countries 0.4%.

d Missing data for education 4.9%.

Of the study cohort, altogether 19.4% were not in the work force. Of these, 8.5% were not registered as working, 7.4% were university students, 2.7% were in early retirement for medical reasons, 0.3% were retired, and 0.6% received home care or were living in a long-term care facility (Table 2). Of the total cohort, 37.6% were in occupations with the possibility to work from home, while 9.8% worked in health and social care, 6.0% as school or preschool staff, 12.8% as essential workers, and 14.5% as blue-collar (but not essential) workers.

Having any prior hospital-recorded diagnosis was uncommon in this population. Those with severe COVID-19 and COVID-19 deaths had much higher rates of any of the comorbid conditions. Of those with no COVID-19 diagnosis, 2.3% were registered with obesity, 2.7% with hypertension, and 2.3% with diabetes, with corresponding proportions among those treated in intensive care of 5.6%, 14.9%, and 14.1%, and among those who died 8.7%, 26.9%, and 23.7%.

## Outcomes

### Any COVID-19

In univariate analyses, male sex and rural or suburban (compared to urban) living were associated with slightly lower RRs for any COVID-19 (largely reflecting the risk of testing positive for COVID-19 (Table S4). In contrast, non-Nordic origin was associated with slightly higher risk. After adjustment for confounders (age, sex, region, Nordic origin and comorbidities), those who had the possibility to work from home, students, persons not working, those in early retirement or retired, and those with home care had lower RRs of COVID-19 infection while living in a long-term care facility was not in itself associated with a higher RR of COVID-19 infection.

**Fig. 1:**
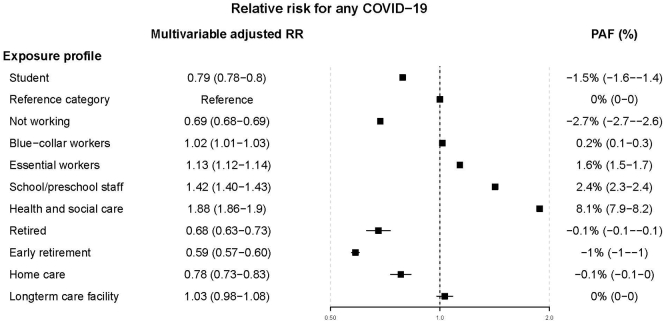
Relative risks and PAFs for any COVID-19 in multivariable-adjusted^1^ models.

Increased adjusted RRs with those being able to work from home as reference were found among essential workers (RR 1·13, 95% CI 1·12–1·14), school and preschool staff (RR 1·42, 95% CI 1·40–1·43), and health and social care staff (RR 1·88, 95% CI 1.86–1·90) compared to the reference group (Fig. 1).

Adjusted for age, sex, region, comorbidities and occupation, the risk of Nordic origin for any COVID-19 was only slightly increased (RR 1·14, 95% CI 1·13–1·14) (Table 3).

**Table 3:** Effect of Nordic origin on risk for any COVID-19, severe COVID-19, intensive care for COVID-19 and death from COVID-19.

Exposure variable	RR	PAF
Any COVID-19		
Nordic origin, no^1^	1.14 (1.13–1.14)	3.1% (2.9–3.3)

Severe COVID-19		
Nordic origin, no^1^	3.02 (2.92–3.12)	31.4% (30.4–32.4)

Intensive care for COVID-19		
Nordic origin, no^1^	3.13 (2.91–3.36)	32.2% (30–34.4)

Death from COVID-19		
Nordic origin, no^1^	2.23 (1.88–2.66)	21.6% (16.5–26.6)

1 Model adjusted for confounders: Age, sex, comorbidities, region and occupation.

### Intensive care and severe COVID-19

**Fig. 2:**
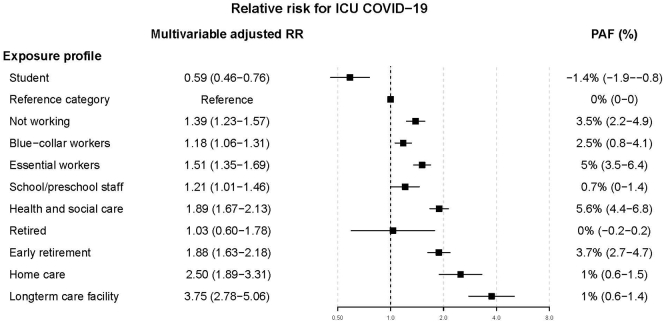
Relative risks and PAFs for need of intensive care for COVID-19 in multivariable-adjusted^1^ models.

Higher age, male sex, and non-Nordic origin were each strong predictors of intensive care for COVID-19 (Table S4). Most comorbidities, notably diabetes and hypertension, were associated with increased risk for intensive care. Compared to occupations with the possibility to work from home, essential workers (RR 1·51, 95% CI 1·35–1·69), school and preschool staff (RR 1·21, 95% CI 1·01–1·46), and healthcare and social care workers (RR 1·89, 95% CI 1·67–2·13) had higher RR of intensive care (Fig. 2). Not working (RR 1·39, 95% CI 1·23–1·57), early retirement (RR 1·88, 95% CI 1·63–2.18), having home care (RR 2·50, 95% CI 1.89–3·31), or being in long-term care (RR 3·75, 95% CI 2·78–5·06) were also associated with higher risk (Fig. 2).

Patterns for severe COVID-19 (Fig. S3) which included all hospitalisations and deaths were similar to for those requiring intensive care. Essential workers (RR 1·35, 95% CI 1·28–1·42), school and preschool staff (RR 1·36, 95% CI 1·27–1·47), and health and social care staff (RR 1·95, 95% CI 1·85–2·06) all had significantly increased multiple-adjusted RR.

In adjusted models, the RRs for non-Nordic origin for ICU and severe COVID-19 were increased (RR 3·13, 95% CI 2·91–3·36 and RR 3·02, 95% CI 2·92–3·12 respectively) (Table 3).

### COVID-19 death

Being born outside a Nordic country was associated with a univariate RR of COVID-19 death of 2·06, 95% CI 1·76–2·42, while intermediate and low education were associated with higher RR: 1·91, 95% CI 1·57–2·33 and 3·11, 95% CI 2·49–3·87 (Table S4). In univariate models, all comorbidities were associated with substantially higher risk of death (Table S4).

**Fig. 3:**
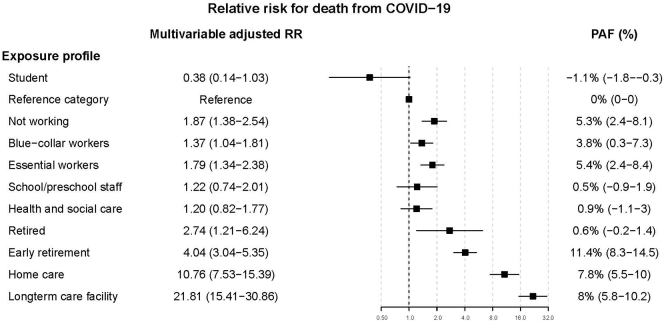
Relative risks and PAFs for COVID-19 death in in multivariable-adjusted^1^ models. ^1^ (adjusted for age, sex, region, comorbidities and Nordic origin).

Compared with the reference group, those not working, in early retirement, having home care, or being in long-term care had multiple-adjusted RRs of 1·87, 95% CI 1·38–2·54, 4·04, 95% CI 3·04–5·35, 10·76 95% CI 7·53–15·39, and 21·81 95% CI 15·41–30·86 (Fig. 3). Essential workers had significantly increased risk of death (RR 1·79, 95% CI 1·34–2·38) compared to the reference group. Blue collar workers (RR 1·37, 95% CI 1·04–1·81) had a slightly elevated risk for death.

After adjustments, non-Nordic origin were associated with a RR of COVID-19 death of 2·23 (95% CI 1·88–2·66) (Table 3).

### Population attributable fractions for COVID-19 outcomes

PAFs were calculated for need of intensive care (Fig. 2). Of the occupational groups, PAFs for health and social care workers, blue-collar, and essential workers taken together contributed 13% (5·6%, 2·5% and 5·0%, respectively), and slightly lower for not working (3·5%) and early retirement (3·7%) while the contribution from school/preschool occupations was very small (<1%). The contribution from home care and long-term care was limited, at about 2%. PAFs for severe COVID-19 which included all hospitalisations and deaths showed a similar pattern to those for ICU care.

With respect to COVID-19 death, those not working, in early retirement, having home care, or being in long-term care had multiple-adjusted PAFs of 5·3%, 11·4%, 7·8%, and 8·0%. Essential workers who had the most increased risk of death compared to the reference group had a PAF of 5·4%.

For any COVID-19 diagnosis, non-Nordic origin had a PAF of only 3%. However, for need of ICU and severe COVID-19 the PAFs were above 30% and 22% for death (Table 3).

## Discussion

In this nationwide study of all persons in Sweden aged 18 to 64 years, covering the first year of the COVID-19 pandemic in Sweden, essential workers, school and preschool workers and health and social care staff all had significantly increased relative risk of a COVID-19 diagnosis and of severe disease compared to a reference category with jobs that allowed working from home. Of the factors associated with exposure to the SARS-CoV-2 virus, working in health and social care was among the most important attributable factors for severe COVID-19 (hospitalisations or death), after non-Nordic origin, and low education, but ranked slightly lower with respect to need of intensive care. Even so, while essential work was associated with increased relative risk of death, teaching or working in a health and social care setting was not. Deaths were mostly attributable to cardiometabolic comorbidities, not being in the workforce, and sociodemographic factors, most notably non-Nordic origin.

Several prior studies in Sweden and elsewhere have found essential occupations to be associated with increased risk of COVID-19 outcomes [5,[14], [15], [16], [17]], but these studies have generally not been concerned with persons outside the work force. A Swedish study found that inequalities in COVID-19 mortality among adults of working age were primarily related to traditional risk factors and not occupation [14], but non-working individuals were not included, nor non-fatal severe outcomes. In the present study, just over half of all deaths (55%) in working-age adults occurred among people in the active work force, and about 5% of the deaths could be attributed to essential work. In contrast to our findings, where school staff were at increased risk of severe COVID-19, a population-based Scottish study found that teachers had no increased risk of hospital admission with COVID-19 and had a lower risk of severe COVID-19 [18]. In health care and education workers, intense space sharing during the workday and high probability of larger numbers of close social contacts were recently demonstrated in an English cohort study, using data from electronic diaries [19], all leading to a high risk of being infected by a coworker.

Few population-based studies have included deaths outside the hospital [20], represented by one in five in the present study, or included non-fatal outcomes [21]. Still, hospitalisations due to COVID-19 in general, and need of intensive care in particular, put a huge burden on already limited and strained health care resources and are important indicators of severe disease. While only 5% of all COVID-19 deaths occurred among adults under 65, they comprised slightly >40% of all hospitalisations and over half of all cases in intensive care in Sweden during the first year, combining the data of the present study and our prior study of persons aged 55 and older [4]. Of all those hospitalised in this comparatively young cohort, 3% died; among those in intensive care, nearly 10% died. Accordingly, the cases needing intensive care attributable to working in health care, being an essential worker, or a blue-collar worker – about 13% – may have been preventable if these individuals were infected at work.

Non-Nordic origin was associated with a 2 to 3-fold increased relative risk of severe disease and death irrespective of adjustments, probably reflecting many issues relevant to higher risk of being infected and of being seriously ill that we could not capture through our registry data. Within the broad job categories that we used some essential workers are more exposed than others to close and frequent contacts with many persons for which service is part of the job, or to being in crowded venues. Still, many immigrants are outside the workforce, and language difficulties, poorer health literacy, and overcrowding could all be important. Sweden displays among the biggest within-country gap in housing depravation inequalities in Europe, as about 30% of all foreign-born persons live in overcrowded households, compared to 9% of Swedish-born persons [22]. Overcrowding, working in highly exposed occupations, with little power to avoid being exposed to infection, together with higher rates of obesity, hypertension, and diabetes, are all more common in areas with many immigrants and low socio-economic status [23], which will have contributed to the high risks observed.

Social gradients in severe COVID-19 have been reported [24], not explained by existing comorbidities. In the present study, education but not income showed a strong independent gradient in COVID- 19 deaths. Deaths in COVID-19 are concentrated among the elderly, most of whom were born in Sweden but had less education compared to those born later. There was a weak positive association between income and all COVID-19 outcomes except death, potentially due to extensive travelling among the more affluent during the Swedish winter school holiday in February 2020 [25,26].

Few studies have provided community-based case fatality rates of infected people in this age range; these were very low, or about 0.06% during the last part of the study period when testing was adequate. Among other studies evaluating younger people, a study of hospitalised patients in the United States showed that 5% were aged 18–34 years [27]. Of these, 2.7% died, which is higher than in the present study, or about 1% among those aged 18–44 years. The higher mortality in the United States, compared to Swedish patients is likely due to differences in patient characteristics, with 25% of the US patients being morbidly obese, and 18% with diabetes, while obesity rates in Sweden are much lower [28]. We know from other studies that obesity and obesity-related disorders are important risk factors for more severe COVID-19 outcomes [29].

In COVID-19 research, absolute and relative risks are often used to describe the effect of risk factors. In this report, we add estimates for PAFs, e.g. the proportional reduction in population disease or mortality would occur if the exposure to a risk factor was reduced to an alternative exposure scenario. While not all factors are modifiable, the concept of attributable risk provides us with information not only about the magnitude of the excess risk, but also about the proportion of the population affected. This can provide additional information on which measures of prevention that are useful from a population perspective. Few studies have evaluated PAFs with respect to COVID-19 outcomes. In our prior Swedish study of persons aged 55 and older in Sweden, we found that home care or living in a long-term care facility were decisive factors for death and severe illness, with over half of deaths attributable to this even after considering multimorbidity [4]. In a retrospective population-based cohort study in the United States, from a database of administrative health claims for members of large health plans from all 50 states, PAFs for COVID-19 death in those younger than 50 were 45% for cardiovascular disease and 31% for diabetes [30], after adjustment for race/ethnicity and a range of other medical conditions. However, social and work-related factors were not considered and the generalizability to any representative background population is uncertain.

Strengths of the present study include the availability of data on multiple medical, occupational, social, and demographic dimensions from a whole nation and data on out-of-hospital deaths. There are also several weaknesses that apply to our study, including that only hospital diagnoses of comorbid conditions were captured, which underestimates the prevalence and importance of obesity and of obesity-related conditions such as hypertension and diabetes. Also, the occupational categorisation that we used is broad and actual presence and conditions at work related to exposure could not be measured. In addition, other unmeasured confounders possibly affect both RR estimates and PAF calculations. For example, COVID-19 tests were used selectively in the first half of 2020 with likely higher testing in some occupations. Additionally, while the use of Poisson regression simplifies the analyses, the effects sizes estimated will represent averages over the whole study period. Of note, however, the already low absolute risks for severe disease and death of many categories in this study should translate into even lower risks in vaccinated persons.

In conclusion, among people of working age in Sweden during the first year of the COVID-19 pandemic, essential workers, school and preschool staff and health and social care staff all had moderately but significantly increased relative risk of a COVID-19 diagnosis and of severe disease, compared to a reference group of people in occupations who were able to work from home, while only essential workers had increased relative risk of COVID-related mortality. A large part of the need of intensive care was attributable to non-Nordic origin and shorter education, with roughly 10% attributable to essential work and work in the health and care sector. Overall, case fatality rates were very low. Deaths were mostly attributable to cardiometabolic comorbidities, not being in the workforce, needing home care or living in a long-term care facility, and other sociodemographic factors.

## Funding

This work was supported by grants from the Swedish state under an agreement concerning research and education of doctors [ALFGBG-966211 (AR), ALFGBG-965885 (MG)]; the Swedish Heart and Lung Foundation [2021-0345]; the 10.13039/501100004359Swedish Research Council [2018-02527 (AR), 2020-05792 (AR), 2021-06525 (AR), 2021-06545 (MG), VRREG 2019-00193 (AR), 2019-00198 (JB), 2019-00245 (MB), 2019-00209 (JL)]; 10.13039/501100009252Science for Life Laboratory from the 10.13039/501100004063Knut and Alice Wallenberg Foundation (2020.0241) (MG), and the 10.13039/501100006636Swedish Research Council for Health, Working Life and Welfare [2021-00304 (MÅ), 2021-00326 (MS)].

## CRediT authorship contribution statement

**Annika Rosengren:** Conceptualization, Funding acquisition, Investigation, Methodology, Project administration, Writing – original draft. **Mia Söderberg:** Conceptualization, Funding acquisition, Methodology, Writing – original draft. **Christina E. Lundberg:** Conceptualization, Investigation, Methodology, Writing – review & editing. **Martin Lindgren:** Conceptualization, Investigation, Methodology, Writing – review & editing. **Ailiana Santosa:** Investigation, Writing – review & editing. **Jon Edqvist:** Investigation, Writing – review & editing. **Maria Åberg:** Conceptualization, Funding acquisition, Writing – review & editing. **Magnus Gisslén:** Conceptualization, Funding acquisition, Methodology, Writing – review & editing. **Josefina Robertson:** Conceptualization, Investigation, Methodology, Writing – review & editing. **Ottmar Cronie:** Methodology, Formal analysis, Software, Validation, Writing – review & editing. **Naveed Sattar:** Investigation, Writing – review & editing. **Jesper Lagergren:** Conceptualization, Funding acquisition, Writing – review & editing. **Maria Brandén:** Conceptualization, Funding acquisition, Methodology, Writing – review & editing. **Jonas Björk:** Conceptualization, Funding acquisition, Methodology, Validation, Writing – review & editing. **Martin Adiels:** Conceptualization, Investigation, Methodology, Formal analysis, Software, Validation, Writing – review & editing.

## Declaration of Competing Interest

The authors declare that they have no known competing financial interests or personal relationships that could have appeared to influence the work reported in this paper.

## Appendix A Supplementary data

Supplementary material Image 1
