# Supplementary material for: COVID-19 in people aged 18–64 in Sweden in the first year of the pandemic: Key factors for severe disease and death
Source: Glob Epidemiol. 2022 Nov 24;4:100095. doi: 10.1016/j.gloepi.2022.100095 (PMC9683858; doi:10.1016/j.gloepi.2022.100095)
Supplement: Supplementary file 1 — Supplementary material [file mmc1.docx]

**Supplement to: COVID-19 in people aged 18-64 in Sweden in the first year of the pandemic: key factors for severe disease and death.**

**Table of Contents**

| **Supplementary methods** | **Page 2** |
| --- | --- |
| **Supplementary table S1. Definitions of baseline comorbidities and procedural codes** | **Page 3** |
| **Supplementary table S2. Main diagnoses used together with a secondary COVID-19 diagnosis.** | **Page 4-5** |
| **Supplementary table S3. Occupational categories** | **Page 6-7** |
| **Supplementary table S4. Univariate risk ratios for COVID-19 outcomes** | **Page 8** |
| **Supplementary figure S1. Directed Acyclic Graph - model with work as main exposure: adjusted for age, sex, region, comorbidities and Nordic origin** | **Page 9** |
| **Supplementary figure S2. Directed Acyclic Graph - model with Nordic origin as main exposure: adjusted for confounders (age, sex) and mediators (region, comorbidities and occupation)** | **Page 10** |
| **Supplementary figure S3. Relative risks for severe COVID-19 (hospitalisation and/or death) in univariate and adjusted models, with the final model adjusted for all variables** | **Page 11** |

**SUPPLEMENTARY METHODS**

# **Definitions of COVID-19 cases**

***ICD 10 codes.*** U07.1 and U07.2 were used to select COVID-19 diagnoses from the patient registry and as an underlying cause of death in the cause-specific death registry

***COVID-19 deaths.*** Deaths were considered as caused by COVID-19 if i) COVID-19 was the underlying cause of death or, ii) COVID-19 was a contributory diagnosis to an underlying diagnosis likely to be COVID-19 related. A list of acceptable underlying diagnoses is presented in **Supplementary Table S2**.

***COVID-19 hospitalizations.*** Hospitalizations were considered COVID-19 hospitalizations if i) COVID-19 was the principal diagnosis or, ii) COVID-19 was a contributory diagnosis with a principal diagnosis likely to be COVID-19 related. A list of acceptable principal diagnoses is presented in **Supplementary Table S2**. If multiple hospitalizations were present for a patient only one hospitalization was accounted for in the following order: COVID-19 as a principal diagnosis, COVID-19 as a contributory diagnosis with an acceptable principal diagnosis. The number of principal diagnoses, acceptable contributory diagnoses and non-acceptable contributory diagnoses are summarized in **Supplementary Table S4.**

***Severe COVID-19.*** Severe COVID-19 was defined as either hospitalization or death by COVID-19 as defined above.

***Date of diagnosis.*** Date of diagnosis was defined in the following order: date of COVID-19-hospitalization (for hospitalized patients), date of first positive test, date of COVID-19 death. All cases registered between January 31, 2020, and February 1, 2021 were included in the study.

**Table S1. Definitions of baseline comorbidities and procedural codes**

| **Diagnosis** | **ICD-9** | **ICD-10** |
| --- | --- | --- |
| Atrial fibrillation | 427D | I48 |
| COPD | 491 | J44 |
| Diabetes | 250 | E10–E14 |
| Dementia | 290 | F00–F09 |
| Heart failure | 428 | I50 |
| Hypertension | 401–405 | I10–I15 |
| Malignancy | 140–208 | C00–C97 |
| Myocardial infarction | 410 | I21 |
| Obesity | 278A, 278B | E65, E66 |
| Stroke (ischemic and haemorrhagic) | 431, 433, 434, 436 | I61–I64 |
| Venous thromboembolism | 415B, 416W, 451 (not 451A) | I26, I80.1-I80.9 |
| **Codes used in severe complication** |  |  |
| **Ventilator** |  | DG002, DG016, DG017, DG018, DG020, DG021, DG022, DG023, DG026, DV023, GBB00,GBB03 |
| High flow oxygen |  | DG028 but not any of above |

**Table S2. Main diagnoses used together with a secondary COVID-19 diagnosis.**

| **Included categories** | **ICD-codes for hospitalization** | **ICD-codes for death** |
| --- | --- | --- |
| **COVID-related symptoms** |  |  |
| Cough | R05 |  |
| Abnormalities of breathing | R06 |  |
| Pain in throat and chest | R07 |  |
| Other symptoms and signs involving the circulatory and respiratory system | R09 |  |
| Dizziness and giddiness | R42 |  |
| Fever of other or unknown origin | R50 |  |
| Headache | R51 |  |
| Malaise and fatigue | R53 |  |
| Syncope and collapse | R55 |  |
| **Upper and lower respiratory tract infections** |  |  |
| Acute nasopharyngitis | J00 |  |
| Acute tonsillitis | J03 |  |
| Acute upper respiratory infections of multiple and unspecified sites | J06 |  |
| Influenza due to other identified influenza virus | J10 |  |
| Other viral pneumonia | J128 |  |
| Viral pneumonia, unspecified | J129 |  |
| Pneumonia due to Streptococcus pneumoniae | J13 |  |
| Bacterial pneumonia, not elsewhere classified | J15 |  |
| Pneumonia due to other specified infectious organisms | J168 |  |
| Pneumonia in diseases classified elsewhere | J17 |  |
| Pneumonia, unspecified organism | J18 |  |
| Unspecified acute lower respiratory infection | J22 |  |
| Coronavirus infection, unspecified | B342 |  |
| Other viral infections of unspecified site | B348 |  |
| Viral infection, unspecified | B349 |  |
| Coronavirus as the cause of diseases classified elsewhere | B972 |  |
| Other and unspecified infectious diseases | B99 | B99 |
| **Respiratory disorders** |  |  |
| Pulmonary embolism | I26 |  |
| Acute respiratory distress syndrome | J80 |  |
| Pulmonary edema | J81 |  |
| Pleural effusion not elsewhere classified | J90 |  |
| Respiratory failure, not elsewhere classified | J96 | J96 |
| Respiratory disorders in diseases classified elsewhere | J99 |  |
| **Obstructive Airway Diseases** |  |  |
| Acute bronchitis due to other specified organisms | J208 |  |
| Acute bronchitis, unspecified | J209 |  |
| Acute bronchiolitis due to other specified organisms | J218 |  |
| Acute bronchiolitis, unspecified | J219 |  |
| Other chronic obstructive pulmonary disease | J44 | J44 |
| Asthma | J45 | J45 |
| **Cardiac diseases** |  |  |
| Viral carditis | B332 |  |
| Chronic ischemic heart disease | I25 | I25 |
| Acute pericarditis | I30 | I30 |
| Pericarditis in diseases classified elsewhere | I32 |  |
| Acute myocarditis, unspecified | I40 |  |
| Myocarditis in diseases classified elsewhere | I41 |  |
| Atrial fibrillation and flutter | I48 | I48 |
| Heart failure | I50 | I50 |
| Abnormalities of heart beat | R00 |  |
| **Kidney disorders** |  |  |
| Acute kidney failure | N17 | N17 |
| Chronic kidney disease | N18 | N18 |
| Unspecified kidney failure | N19 | N19 |
| **Electrolyte disorders** |  |  |
| Other disorders of fluid, electrolyte and acid-base balance | E87 | E87 |

**Table S3. Occupational categories**

| **Occupation** |  |
| --- | --- |
| School and pre-school teachers | 2320, 2341, 2342, 2343, 2351, 3423, 5311, 5312 |
| Health and social care workers | 2211, 2212, 2213, 2219, 2221, 2222, 2223, 2224, 2225, 2226, 2227, 2228, 2231, 2232, 2233, 2234, 2235, 2239, 3411, 5321, 5322, 5323, 5324, 5325, 5326, 5330, 5341, 5342, 5343, 5349, 2241, 2242, 2260, 2271, 2272, 2273, 2281, 3212, 3213, 2282, 2283, 2284, 3250, 5350, 3230 |
| Essential workers | 1711, 1712, 1721, 1722, 1731, 1732, 1741, 1742, 1791, 1792, 2661, 2662, 2663, 2669, 2671, 2672, 3412, 3333, 3353, 3441, 3451, 3452, 4117, 4224, 4225, 5111, 5112, 5113, 5131, 3230, 5132, 5141, 5142, 5143, 5144, 5149, 5151, 5152, 5221, 5222, 5223, 5224, 5225, 5226, 5227, 5230, 9111, 9411, 9412, 9413, 3351, 3360, 5412, 5413, 4420, 8321, 8329, 8331 |
| Blue-collar jobs | 3121, 3122, 4321, 4322,  7xxx  8xxx, exlcuding 8321, 8329, 8331  9xxx, excluding 9111, 9411, 9412, 9413 |
| Persons in occupations compatible with being able to work from home and/or with few social contacts | 1111, 1112, 1113, 1120, 1211, 1212, 1221, 1222, 1230, 1241, 1242, 1251, 1252, 1291, 1292, 1311, 1312, 1321, 1322, 1331, 1332, 1341, 1342, 1351, 1352, 1361, 1362, 1371, 1372, 1380, 1411, 1412, 1421, 1422, 1511, 1512, 1521, 1522, 1531, 1532, 1540, 1611, 1612, 2111, 2112, 2113, 2114, 2121, 2122, 2131, 2132, 2133, 2134, 2135, 2141, 2142, 2143, 2144, 2145, 2146, 2149, 2161, 2162, 2163, 2164, 2171, 2172, 2173, 2179, 2181, 2182, 2183, 2250, 2311, 2312, 2313, 2314, 2319, 2330, 2411, 2412, 2413, 2414, 2415, 2419, 2421, 2422, 2423, 2431, 2432, 2511, 2512, 2513, 2514, 2515, 2516, 2519, 2611, 2612, 2613, 2614, 2615, 2619, 2621, 2622, 2623, 2641, 2642, 2643, 2651, 2652, 2653, 2654, 2655, 3111, 3112, 3113, 3114, 3115, 3116, 3117, 3119, 3151, 3152, 3153, 3154, 3155, 3211, 3214, 3215, 3240, 3311, 3312, 3313, 3314, 3321, 3322, 3323, 3324, 3331, 3332, 3334, 3335, 3339, 3341, 3342, 3343, 3352, 3354, 3355, 3359, 3431, 3432, 3433, 3439, 3449, 3511, 3512, 3513, 3514, 3515, 3521, 3522, 4111, 4112, 4113, 4114, 4115, 4116, 4212, 4221, 4222, 4223, 4226, 4323, 4410, 4430, 5161, 5169, 5414, 5419, 6111, 6112, 6113, 6121, 6122, 6129, 6130, 6210, 6221, 6222 |
| University students | Main income from student loan or registered as a student |
| Early retirement | Main income from early retirement for medical reasons |
| Retired | Main income from retirement pension |

## **Table S4. Univariate risk ratios for COVID-19 outcomes**

| **Heading** | **Any COVID-19** | **Severe COVID-19** | **ICU COVID-19** | **COVID-19 death** |
| --- | --- | --- | --- | --- |
| **Age group** |  |  |  |  |
| 18 to 44 | 1.17 (1.16-1.17) | 0.21 (0.2-0.22) | 0.12 (0.11-0.13) | 0.05 (0.04-0.07) |
| 45 to 54 | 1.23 (1.22-1.25) | 0.63 (0.61-0.66) | 0.52 (0.48-0.56) | 0.29 (0.24-0.35) |
| 55 to 64 (ref) | 1 | 1 | 1 | 1 |
| **Male** | 0.84 (0.83-0.84) | 1.51 (1.47-1.56) | 2.30 (2.14-2.48) | 2.48 (2.08-2.95) |
| **Baseline** |  |  |  |  |
| Diabetes | 1.04 (1.02-1.06) | 5.47 (5.22-5.74) | 7.08 (6.43-7.8) | 13.45 (11.18-16.18) |
| Hypertension | 0.95 (0.93-0.97) | 5.12 (4.89-5.36) | 6.39 (5.81-7.02) | 13.43 (11.25-16.04) |
| AF | 0.98 (0.95-1.02) | 4.04 (3.65-4.46) | 3.81 (3.03-4.79) | 9.19 (6.44-13.11) |
| Demens | 0.97 (0.87-1.09) | 6.77 (5.33-8.6) | 3.94 (1.97-7.89) | 61.50 (40.19-94.11) |
| COPD | 0.89 (0.86-0.93) | 4.65 (4.23-5.12) | 4.74 (3.85-5.83) | 14.30 (10.65-19.19) |
| HeartFailure | 0.86 (0.82-0.91) | 6.71 (6.02-7.48) | 7.81 (6.25-9.76) | 23.24 (16.93-31.89) |
| Cancer | 0.99 (0.96-1.02) | 2.34 (2.12-2.58) | 2.22 (1.78-2.77) | 6.17 (4.46-8.53) |
| Obesity | 1.18 (1.16-1.2) | 2.33 (2.18-2.5) | 2.56 (2.21-2.96) | 4.10 (3.10-5.42) |
| VTE | 1.06 (1.03-1.08) | 3.37 (3.10-3.65) | 3.68 (3.1-4.37) | 6.09 (4.42-8.39) |
| MI | 0.9 (0.86-0.94) | 4.29 (3.87-4.75) | 5.75 (4.72-7.0) | 10.44 (7.36-14.83) |
| Stroke | 0.84 (0.80-0.88) | 3.49 (3.10-3.92) | 3.71 (2.9-4.76) | 9.65 (6.65-14.01) |
| **Non-Nordic origin** | 1.12 (1.11-1.12) | 2.84 (2.76-2.93) | 2.90 (2.71-3.1) | 2.06 (1.76-2.42) |
| **Education** |  |  |  |  |
| <=9 years | 0.86 (0.85-0.86) | 1.72 (1.65-1.79) | 2.01 (1.84-2.21) | 3.11 (2.49-3.87) |
| 10-12 years | 1 (1.00-1.01) | 1.27 (1.23-1.32) | 1.42 (1.31-1.54) | 1.91 (1.57-2.33) |
| University (Ref) | 1 | 1 | 1 | 1 |
| **Region** |  |  |  |  |
| Suburban | 0.90 (0.89-0.91) | 0.69 (0.64-0.73) | 0.68 (0.59-0.79) | 0.55 (0.39-0.8) |
| Rural | 0.70 (0.70-0.71) | 0.57 (0.54-0.6) | 0.54 (0.48-0.61) | 0.47 (0.35-0.64) |
| Urban (Ref) | 1 | 1 | 1 | 1 |
| **Exposure level** |  |  |  |  |
| Student | 0.84 (0.83-0.85) | 0.47 (0.42-0.52) | 0.36 (0.28-0.46) | 0.16 (0.06-0.43) |
| Reference category^1^ | 1 | 1 | 1 | 1 |
| Not working | 0.74 (0.73-0.75) | 2.04 (1.94-2.15) | 2.08 (1.85-2.33) | 2.46 (1.84-3.3) |
| Blue-collar workers | 0.97 (0.96-0.98) | 1.27 (1.21-1.34) | 1.55 (1.4-1.72) | 1.79 (1.36-2.35) |
| Essential workers | 1.16 (1.15-1.17) | 1.44 (1.37-1.52) | 1.59 (1.42-1.77) | 1.77 (1.33-2.35) |
| School/preschool staff | 1.45 (1.43-1.46) | 1.18 (1.09-1.27) | 0.9 (0.75-1.08) | 0.89 (0.54-1.46) |
| Health and social care | 1.92 (1.9-1.93) | 1.96 (1.87-2.06) | 1.69 (1.5-1.9) | 1.04 (0.71-1.52) |
| Retired | 0.60 (0.56-0.65) | 1.96 (1.52-2.52) | 2.05 (1.18-3.54) | 6.92 (3.05-15.69) |
| Early retirement | 0.58 (0.56-0.59) | 3.51 (3.29-3.74) | 4.12 (3.6-4.72) | 10.54 (8.07-13.77) |
| Home care | 0.79 (0.74-0.85) | 9.88 (8.81-11.08) | 9.28 (7.14-12.07) | 61.07 (44.31-84.18) |
| Longterm care facility | 1.07 (1.01-1.12) | 6.01 (5.27-6.86) | 6.05 (4.52-8.11) | 49.07 (35.53-67.79) |

**^1^ possible to work from home**

**
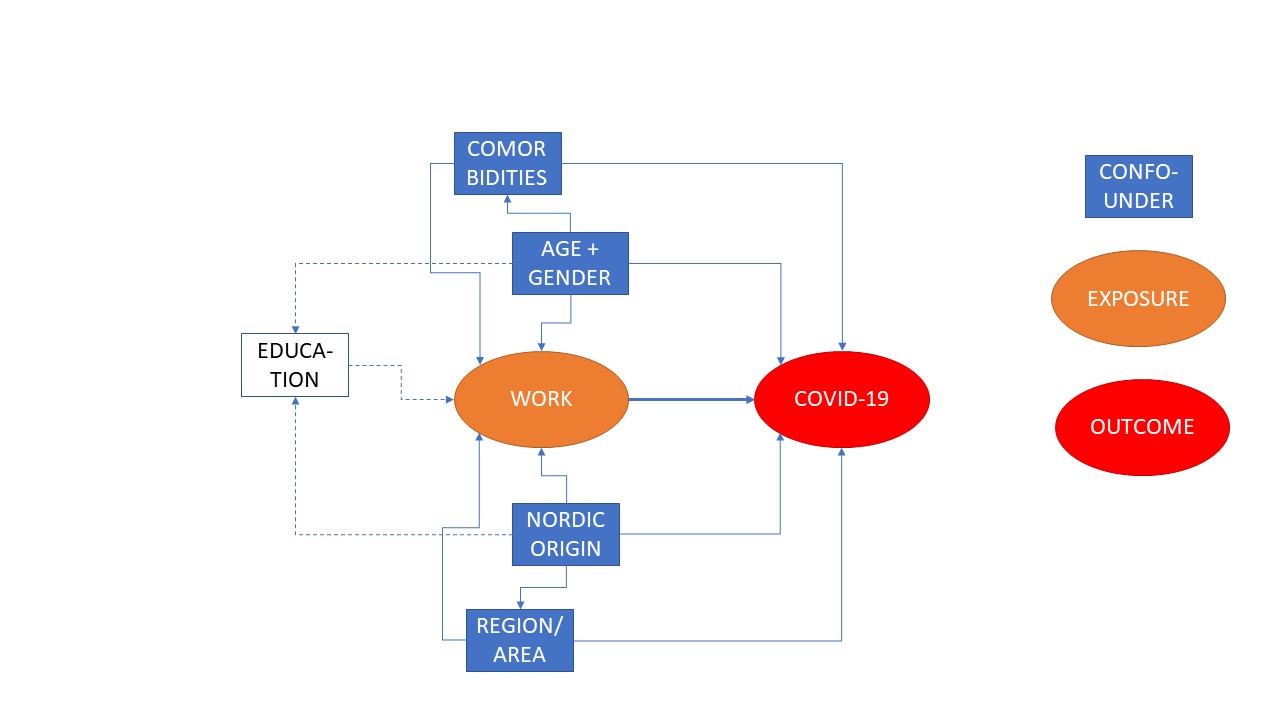
**

**Figure S1. Directed Acyclic Graph - model with work as main exposure: adjusted for age, sex, region, comorbidities and Nordic origin**

**
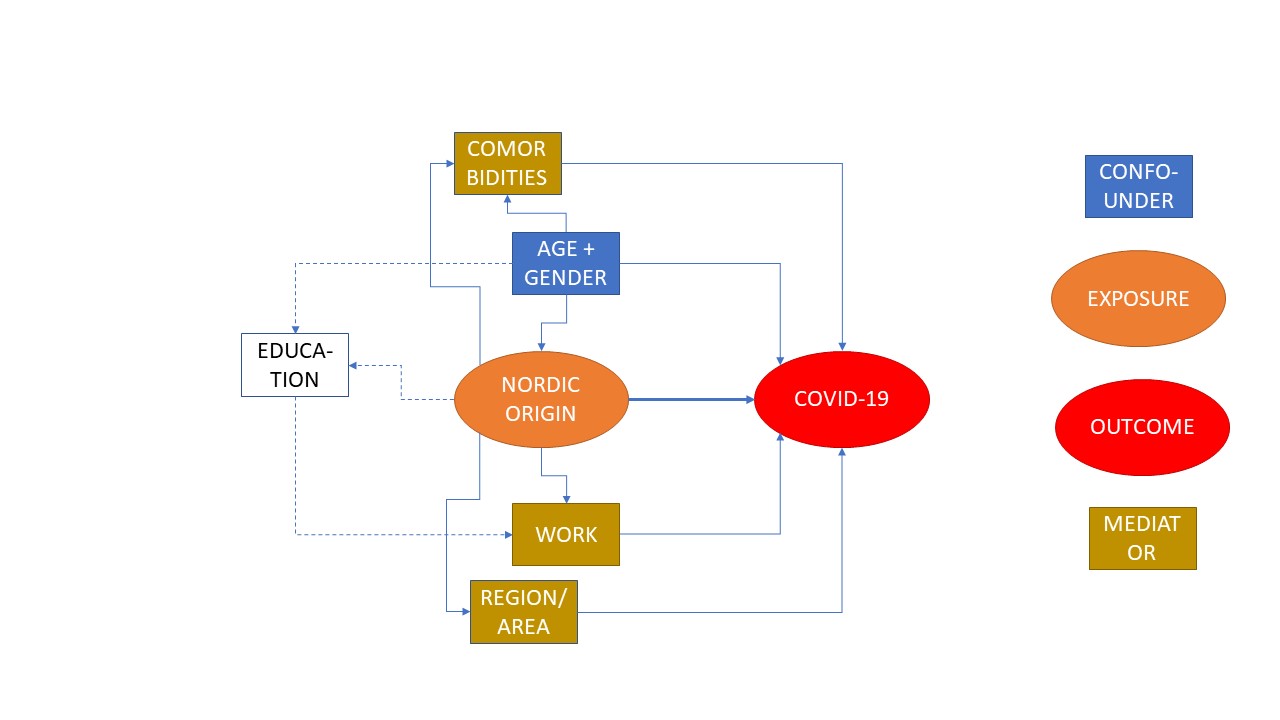
**

**Figure S2. Directed Acyclic Graph - model with Nordic origin as main exposure: adjusted for confounders (age, sex) and mediators (region, comorbidities and occupation)**


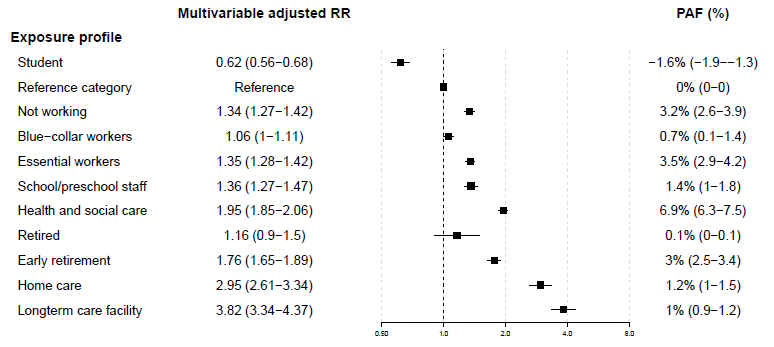


## **Figure S3. Relative risks and PAFs for severe COVID-19 (hospitalisation and/or death) in multivariable-adjusted models (adjusted for age, sex, region, comorbidities and Nordic origin)**
